# Perityphlitis and Its Surgical Treatment1Read before the Hornellsville Medical and Surgical Association, December 2, 1889.

**Published:** 1890-04

**Authors:** Herman Mynter

**Affiliations:** Professor of Surgery, Niagara University; 195 Franklin Street


Buffalo Medical and Surgical Journal

VOL. XXIX. APRIL, 1890. No. 9.

### Original Communications.

### PERITYPHLITIS AND ITS SURGICAL TREATMENT.[1]

By HERMAN MYNTER, M. D.,

Professor of Surgery, Niagara University.

TEN years ago, (September 2, 1879,) I read a paper before the Buffalo Medical Association on Perityphlitis, which was published in the October number, 1879, of the BUFFALO MEDICAL AND SURGICAL JOURNAL. I called the attention of the Association to the operative treatment, then new, of this disease as advocated by the late Dr. Gurdon Buck, and described its pathology, diagnosis, prognosis, and treatment.

In discussing the pathology, I followed the nomenclature of Prof. With, of Copenhagen, who described (1) a peritonitis appendicularis adhesiva, in which the ulceration in the appendix vermiformis goes so deep that the peritoneal covering is affected and adhesions are formed; (2) a peritonitis appendicularis localis, characterized by local peritonitis and primary abscess; and (3) a peritonitis appendicularis universalis, in which we have diffuse peritonitis by perforation into the peritoneal cavity.

The cases belonging to the first division, were those with obscure symptoms, local tenderness in right ileo-cecal region, a little vomiting and general ill-feeling for a few days. I stated that they recovered generally promptly by rest, opium, poultices, and avoiding cathartics, but that they frequently relapsed in course of time and might then be followed by the more severe forms. The second form, peritonitis appendicularis localis, I described as characterized by local abscess, generally and primarily intra-peritoneal, but on account of adhesions in reality extra-peritoneal, and extending downwards toward Poupart's ligament, above which, in course of time, they might be and ought to be opened by operation. I have seen and treated six such cases suc-

1. Read before the Hornellsville Medical and Surgical Association, December 2, 1889.

cessfully. For a time the treatment is rest, opium, poultices and no cathartics, and the operation ought to be performed as early as possible to avoid perforation into the abdominal cavity. The third form, peritonitis appendicularis universalis, could either start as such if the perforation took place before adhesions had formed, or by secondary rupture of a well-developed abscess. In regard to these cases, I stated ten years ago that they almost universally terminated fatally in a few days, that no treatment was of any avail, but I expressed a belief (page 122,) that the time would come when in such cases we would open the abdominal cavity and ligate the appendix vermiformis.

During the past ten years a great deal has been written about perityphlitis and its treatment, and it is now almost universally acknowledged that this is distinctly a surgical disease which can only be treated by surgical means, particularly in its most severe forms, where we have either a circumscribed abscess or a diffuse peritonitis.

The three divisions,—adhesive, circumscribed, and diffuse peritonitis,—are still recognized as the different forms, and surgeons differ now very little in regard to the treatment of the second class, circumscribed abscess. It ought to be opened by direct incision as early as it is possible without opening the abdominal cavity. It is in regard to the third form, diffuse peritonitis, that there still is some doubt about the treatment.

The question has been discussed by the leading medical associations of England, the Continent, and America, and is still being discussed; the medical journals are full of reports of cases of successul operations, and it is acknowledged, by all surgeons at least, that this disease can only successfully be treated by surgical means, while physicians yet are loth to acknowledge that it has passed into the domain of surgery. In Buffalo, for instance, a young physician, not long ago, was allowed to die in five days from perforative peritonitis in the hands of an old and most distinguished physician, who discouraged all thoughts of operative interference and relied upon poultices and opium. The post-mortem showed diffuse peritonitis from perforation of the appendix and satisfied the attending physician, but too late, that nothing but an early operation could have been of any avail.

Compare the results of a few years ago when the disease was treated expectatively with those of to-day!

In the discussion of my paper ten years ago the late Dr. Rochester, for instance, stated that he had treated twenty-three cases, seventeen of which were fatal, a mortality of seventy-four per cent.

Krafft gives a statistic of 106 cases (probably an old one, as only eight were operated, gathered together through years,) with a mortality of eighty-four—seventy-nine per cent. I myself have, during the last few years, treated twelve cases, all of which recovered, six after operation. In the following pages I shall give a short review of the opinions of the best known surgeons, both here and abroad, in regard to disputed points.

Bull (*Transactions of the American Surgical Association*) considers perityphlitis an inflammation of either cecum or appendix with their peritoneal covering or the cellular tissue in the iliac fossa. He considers it impossible to distinguish between an inflammation of the cecum and the appendix. He, as do most writers, thinks ulceration leading to perforation more frequent in the appendix than in the cecum, but calls the attention to the fact that catarrh of the cecum, in which the appendix participates, is of frequent occurrence too. I suppose the truth is that it may start in either, and that the catarrh by dilatation may favor the entrance of fecal matter, which again may form a concrement with consecutive ulceration, and that perityphlitis from whatever cause always is accompanied with inflammation of the neighboring peritoneum. Bull distinguishes between a catarrhal perityphlitis tending toward recovery, but then leaving behind adhesions to the parietal peritoneum, the intestines or the omentum, and a suppurative perityphlitis, which either may be spreading (diffuse peritonitis) or limiting (circumscribed peritonitis), followed by extra-peritoneal abscess.

A catarrhal perityphlitis may go on to a suppurative form too. Krafft (*Volkman's Klinische Vortrage*, Jan., 1889) on the other hand considers resolution impossible and believes that there is always a pus focus left, which may not give any symptoms but which may again start up at any time. He mentions a statistic of 106 cases, in eighty-four of which an autopsy was made, and in each an ileo-cecal abscess was found. Operation was performed in eight cases and in each an ileo-cecal abscess was found. The remaining fourteen cases opened spontaneously into cecum and elsewhere. Judging from the few operations performed, and the great mortality, the statistic is probably more than ten years old. It is scarcely possible to get such a statistic together now. That resolution may and does take place is conclusively shown by my countryman, Dr. Toft of Copenhagen, who in thirty-five per cent. of all post-mortem examinations found residua, in the form of adhesions, of perityphlitic inflammation. It shows, too, the frequency of the trouble.

Mikulicz, of Königsberg (*Annals of Surgery*, October, 1889), distinguishes two forms of perforative peritonitis, which are essentially distinct. The first form, diffuse septic peritonitis, results when a large quantity of intestinal contents suddenly pours into the abdominal cavity through a large perforation. The resulting peritonitis is characterized by sanguino-serous or purulent, putrid, thin, fluid exudation, injected peritoneum, at times covered by thin fibrinous deposit. Extensive adhesions are lacking. Laparatomy is always indicated in order to find and suture the opening and disinfect the peritoneum. In the second form, which he calls progressive fibro-purulent peritonitis, the peritoneum is at first only affected in the immediate vicinity of the perforation, a fibro-purulent exudation is formed which prevents by adhesions the infection of the whole peritoneal cavity. The process spreads and incapsulated pus foci are formed between the glued intestines.

The treatment of this form consists in opening each intra-peritoneal focus separately and protecting most carefully the adhesions. Laparatomy is, therefore, absolutely contra-indicated. Mikulicz reports two successful cases of the latter; in one six intraperitoneal pus cavities were opened through three incisions; in another three pus cavities by three incisions. The openings were made at different times as the existence of the pus foci became evident.

Treves (discussion in the British Medical Association, August, 1889,) does not believe in a catarrhal form of perityphlitis. If catarrh only is present we have colitis, not typhlitis; the latter is always produced by ulceration, and the symptoms occur first when the ulcers have extended to the outer wall. Perforation of the cecum primarily he considers rare. The milder forms he thinks are caused by peritonitis of the cecum, the graver by disease of the appendix.

The late Dr. Sands (*New York Medical Journal*, February, 1888,) makes the usual three sub-divisions but mentions besides as a fourth division, obscure cases with slowly progressing symptoms, moderate pain in cecal region, little tenderness and no swelling, with little or no fever. After a time they grow worse with increasing tenderness, meteorrhismus, collapse, and death. Post mortem shows perforation and gangrene, insufficient adhesions and a slowly progressing septic peritonitis with pus and fecal matter between the coils. I am inclined to believe that these cases, of which I never saw any, represent secondary perforation into the abdominal cavity of a primarily limiting or circumscribed perityphlitis.

The question whether a perityphlitic abscess is intra- or extra-peritoneal has been debated and debated again. I do not see the reason for any disagreement on this point. Both cecum and the appendix are, according to Bull and others, always completely invested with peritoneum. An abscess which starts in the appendix must necessarily in the beginning be intra-peritoneal, limited by adhesions. If the adhesions are strong, and exudations continue to be deposited so that perforation into the abdominal cavity is prevented, the parietal peritoneum will become perforated, and the pus is then in the retro-peritoneal tissue in the iliac fossa,—an extra-peritoneal abscess. After such an abscess has been opened, it is often possible to feel the agglutinated coils of the intestines that form the roof and anterior wall.

Krafft believes perforation of the cecum always to be secondary, the abscess perforating into the cecum instead of elsewhere.

Robert Weir (discussion in New York Surgical Society, April, 1889,) rather conclusively proves the intra-peritoneal origin. He had found in one hundred autopsies general suppurative peritonitis fifty-seven times, circumscribed abscesses thirty-five times (in thirteen of which general peritonitis also was present) and extra-peritoneal abscess only in four cases. In each of these four cases there was a large, ragged opening, showing that an ordinary necrotic process of the peritoneal wall had made the abscess extra-peritoneal.

While I therefore agree that these abscesses necessarily are intra-peritoneal in the start, I, on the other hand, must agree with the late Dr. Sands that, if circumscribed in the iliac region, they always are, for all practical purposes at least, extraperitoneal, take the course of all abscesses in this region and must be opened by an extra-peritoneal operation, those of progressive fibro-purulent peritonitis of Mikulicz alone excepted. These are always, even when circumscribed, intra-peritoneal.

A few words may be said about the statistics of relapses, perforations, and fecal concretions which have been gathered during the last ten years. A person who has recovered from an attack of perityphlitis is ever after in danger of a relapse, which may be either mild or the most severe form of perforative peritonitis. Krafft mentions a statistic of 106 cases, of which twenty-four—twenty-three per cent.—had had previous attacks, generally one to three years previously, in one case twenty years previously. Treves mentions one case who had had fourteen attacks and had been in bed twelve months, and Lawson Tait one who had three attacks inside six months. In regard to perforations, Matterstock found perforations in 132 of 146 cases, Fenwick in 113 of 129—ninety and eighty-six per cent. The perfora tion is usually at the free end, but may be circular and, as Krafft says, so to speak amputate the appendix. In Matterstock's 146 cases fecal concretions were found sixty-three times, a foreign body nine times. In Krafft's 106 cases thirty-six fecal concretions and four foreign bodies were discovered. Only small bodies can enter on account of Gerlach's valve. A cherry pit can enter only with difficulty, a plum stone not at all. Sedentary habits and constipation are considered predisposing.

In regard to symptoms I shall be brief, as they are well known. In catarrhal inflammation, dull pain and tenderness in the right iliac region are predominant, besides loss of appetite, nausea, slight vomiting, constipation or diarrhea. If severe chills and fever follow, suppuration may be suspected, but even then the symptoms may disappear. I lately saw such a case in a lawyer, thirty-nine years of age, in which the disease was ushered in with severe chills, high fever, vomiting, and tenderness, with tympanitic percussion. A distinct tumefaction and resistance was felt the third day, yet all the symptoms disappeared inside a week.

The tumor must not be confounded with the cecum filled with feces and extending upwards toward the ribs.

If an abscess is forming, the fever will continue but be less intense, the temperature ranging between 101° and 103°. The vomiting stops, the tumor is felt more and more plainly, extending downwards toward Poupart's ligament; but the tenderness, which during the first twenty-four hours is not always confined to the iliac region but may be present over almost the whole abdomen, is now present only here, the rest of the abdomen being soft and not tender. Then comes a rather anxious time for the surgeon, he being doubtful whether to operate immediately or not, as more severe symptoms may suddenly occur, indicating perforation of the abscess.

The earlier the operation is done the more difficult it is, as the peritoneum is not pushed aside and lifted away from the iliac fascia by the abscess. In a recent case, operated on the seventh day, I made the usual incision down to the fascia transversa. I then introduced the exploring needle, and meeting pus I carefully incised with the needle as a guide. I was rather surprised by getting prolapse of the omentum, having opened the peritoneal cavity. I closed the opening with catgut sutures, went in one-half inch lower, lifted the peritoneum up for about two inches and succeeded in opening the abscess from behind. My patient recovered without mishap.

If perforation occurs primarily or secondarily the chill, pain, and vomiting are followed by collapse and the tenderness spreads over the whole abdomen. Tympanitis sets in, fecal vomiting occurs, prostration increases, and death follows in from three to five days. Bull mentions as diagnostic signs of spreading peritonitis: Continuance of pain, vomiting, constipation, fever and rapid pulse, abdomen slightly swollen, walls rigid and resisting, but tenderness most marked in iliac region where there is found more resistance and tympanitic percussion. On the other hand we meet cases where the vomiting stops, the fever is slow, the pulse less rapid, yet the general tenderness indicates spreading peritonitis.

McDougall (*Lancet*, September, 1888) gives the following diagnostic symptoms of perforation: Pain is more sudden and agonizing and often fixed at a distance, as the epigastrium, umbilicus, bladder, and nervus cruralis. Vomiting is not continuous. Temperature lower than in circumscribed perityphlitis, seldom above 101°, pulse higher, above 120; rapid formation of iliac tumor.

Krafft mentions flexion of the hip-joint as characteristic of perityphlitis. I disagree with him on that point. I never saw flexion (contraction of the ileo-psoas muscle) in perityphlitis, and see no reason why it should occur. The strong fascia iliaca is between the abscess and the muscle, otherwise the point of perforation of the abscess, if left to itself, would be down on the femur below Poupart's ligament. Contraction of the psoas muscle, in short, occurs only when the muscle either is actually inflamed (acute psoitis) or perforated and infiltrated with pus from a cold abscess depending upon necrosis of pelvis, caries of spine, etc.

When we, lastly, consider the treatment, then there is little difference of opinion in regard to adhesive perityphlitis. All authors agree now that the adhesive peritonitis is best treated by absolute rest, absolute diet, avoidance of all cathartics, and opium in sufficient amount to prevent peristaltic movements. My old preceptor, Prof. With of Copenhagen, kept his patients constipated even for three weeks, and I have followed his example in my cases, the only disagreeable result being that I sometimes have had to deliver by manipulations the old and hard scybalæ. I do not use morphia, but the common tincture of opium. I do not believe morphia retards or prevents peristaltic movements as well as opium.

We see yet, occasionally, these cases treated with cathartics and I cannot strongly enough discourage this practice, which I consider a most dangerous one.

We probably always have first a dilatation of the appendix and its opening into the cecum, and consequently the contents of the bowels enter with greater ease. The appendix has a large absorbent surface so that the fluid is absorbed while the solid parts are left and form concrements. It stands to reason that, under such circumstances, cathartics are injurious, as we succeed only in making the contents of the bowels thinner, so that they easier can enter the dilated or inflamed appendix, increase the concrements and favor the ulceration. Baldy's method, the use of strong cathartics in peritonitis, has its use, and is no doubt of benefit in septic peritonitis following laparatomies, for instance, but is, in my opinion, distinctly dangerous in cases in which we have perforation of a hollow viscus or threatening perforation, as in perityphlitis.

Neither is there any difference of opinion in regard to the treatment of circumscribed perityphlitis except in some minor points, as, for instance, the direction of the incision, the time when it ought to be performed and the like Bull advises to operate as early as possible (one week) by incision parallel with and a finger's breadth above Poupart's ligament—Gurdon Buck's method. The incision passes gradually through the different layers till the transversalis fascia is reached. Note whether the muscles are edematous, as it indicates that the abscess is near. An exploring needle may now be used, and, pus being found, incision may be done with the needle as a guide; or, what is safer, the deeper tissues may be torn through with two forceps till pus is found. In one case I got prolapse of the omentum by incising on the needle. No operation is complete till pus is found, although occasionally the abscess has not been found.

It is recommended, then, to plug the opening with iodoform gauze, and the abscess will discharge itself through the opening later. Concretions having been removed if present, a large drainage-tube is introduced, the abscess cavity syringed out with an antiseptic fluid, and an antiseptic bandage applied. All symptoms disappear immediately and the wound heals generally in three weeks. Nothing is seen or felt of the appendix as a rule. It is probably completely obliterated, as there is no case on record in which relapse occurred after a successful operation of a circumscribed perityphlitic abscess.

In regard to the question when the operation should be done: Sands advises to operate between one and two weeks; Bull as soon as you can (one week). My experience is that the earlier the operation is done the more difficult it is. The peritoneum is not pushed away from the iliac fascia and we run the risk of opening the peritoneal cavity, as happened to me in one case. The abscess must then be attacked from behind. The later it is done, the easier is it, but for days we run the risk of secondary perforation into the abdominal cavity. If the tumor has extended so far down that you can feel its lower margin just above the Poupart's ligament, you can operate without fear. If the lower margin is an inch above Poupart's ligament I should prefer to wait, unless serious symptoms occur.

While there is, as we have seen, little disagreement in regard to the treatment of adhesive and circumscribed perityphlitis, it is different when we come to consider the treatment of perforative peritonitis in its several forms. Ten years ago I predicted that in such cases we would shortly make laparatomy, extirpate the appendix and cleanse the abdominal cavity, and this treatment has now been recognized by all surgeons as the only one which offers any chance of recovery. There is still some diversity of opinion in regard to the best method of operation, the time for doing it and how to treat the appendix; but the universal comment, in fatal cases, is that the operation was done too late. Mikulicz was the first to perform laparatomy for a non-traumatic perforative peritonitis in 1880. Bull has made laparatomy in six cases for supposed perforation, two of which died. His earliest operation was done in thirty-six hours, the latest in five days. He advises laparatomy at once in spreading peritonitis, whether due to a primary or secondary perforation. He prefers a vertical or slightly oblique incision, three or four inches long, and starting one inch above the middle of Poupart's ligament. Having opened the peritoneum, he separates the adhesions to the omentum and bowels, evacuates the fecal matter and concretions, taking particular care to protect the abdominal cavity with sponges. If he can find the appendix he advises to ligate and promptly remove it; if the cecum is perforated, he would suture the wound; if the omentum is much infiltrated, he advises to resect it. After carefully cleansing the abdominal cavity, he plugs the wound with iodoform gauze, having first introduced a large drain to the bottom of the fossailiaca.

McDougall prefers to wait till the fourth or fifth day in order to let adhesions form. If symptoms of collapse follow the perforation he lets the patient rally first. He prefers a median incision, complemented, if necessary, with a lateral opening in the iliac region. He may possibly be able to cleanse the abdominal cavity better with a median incision, but it will be more difficult to find and treat the appendix. Robert Weir has had a successful operation inside twenty-four hours after perforation, but has never seen any recover after the peritonitis has become general. Most members of the New York Surgical Society were in favor of delaying the operation till pus had formed, and expressed themselves against immediate operation. Yet the more and more frequent reports of successful early operations speak volumes against delay. Dr. Jacobus, for instance, reports a successful case of early operation in the New York *Medical Record* of February, 1889; Dr. Sands, another one, a boy eleven years of age, performed inside forty-eight hours. (New York *Medical Journal*, February, 1888.) Sands used a vertical incision and found spreading peritonitis without limiting adhesions. A fecal concretion was discovered and removed. The appendix was found ulcerated but not gangrenous, and was left in situ. The wound was treated openly with iodoform gauze. Sands thinks that operation cannot be done too early and, to be successful, must be done before the septic peritonitis has become general. The American surgeons are the ones who have taken the lead in advocating early operations.

Sonnenburg (Discussion in the Surgical Association of Berlin in July, 1889,) thought operation useless if we had diffuse peritonitis. He proposed in doubtful cases to make an incision down to the peritoneum, and then again examine by palpation. If he then could not feel the abscess he would plug the wound with iodoform gauze and examine next day again, possibly with the exploring needle. He, so to speak, advised operation *en deux temps.* I can see no advantage in this proposition. The opening of the peritoneal cavity, if done with proper precautions, would add no danger to the operation, and would at once inform us of the conditions and of the means to overcome them. Most authors agree that operation is useless when the peritonitis has become general. Why then allow it to become general? I myself can see no prospect of success except in early and immediate operation, at most waiting till the patient has rallied from the collapse. In one of my cases, as mentioned, I unintentionally opened the abdominal cavity and got prolapse of the omentum, but my patient recovered nevertheless.

Lastly, one important question is left: what to do in relapsing attacks of perityphlitis? That a mild previous attack is no guarantee that later attacks will be equally mild, is already mentioned; also, that a patient with perityphlitis is ever after in danger of relapse. The question has, therefore, naturally been asked if it is not less dangerous to extirpate the appendix while the patient is well or convalescent, than to let him run the risk of a further attack about the severity of which we can have no opinion.

A positive cure, Krafft says, free from relapses is only possible with operative treatment. He believes that in a few years every perityphlitic abscess will be operated upon, even after the disappearance of all symptoms, and the appendix ligated and cut off. Bull considers it still doubtful whether we in relapsing cases ought to make laparatomy and remove the appendix.

It is our English cousins who have been first to carry this idea into execution, and particularly that master of laparatomy, Lawson Tait. The *British Medical Journal*, of October 5, 1889, contains the report of a case of recurrent perityphlitis successfully treated by abdominal section by Mr. Tait. The patient, twenty-seven years of age, had suffered from repeated attacks of perityphlitis for six months, three in all, the last one being the most severe. During each attack the characteristic egg-shaped tumor had been felt during the acute attack, and was still distinctly marked. Incision was made over the cecum, three inches long and one inch from the anterior superior spine of the ileum, and a suppurating cavity opened on the outside of the cecum. The appendix was discovered swollen to three times its size. It was split open about half an inch from its free end and some purulent fluid evacuated. A foreign body, felt in the appendix higher up, was pushed into the cecum with a catheter. Although the operation had been made extra-peritoneally, the abdominal cavity was opened during the manipulations but the opening was closed with sutures. The appendix was not removed but a drainage-tube was introduced into it and left in position for a couple of days. The patient recovered without bad symptoms.

In similar cases Lawson Tait has twice removed the appendix, finding it thickened, swollen, and suppurating; but he thinks the risk increased by this proceeding and the removal unnecessary. He would prefer opening the appendix and draining it. Treves mentions one patient who had had fourteen attacks and been in bed twelve months; he, too, recovered. He also is in favor of operating between the attacks in cases of relapsing perityphlitis. I have so far seen no case in which I would have advised this proceeding. Although the percentage of relapse is high—twenty-three per cent.—it is in my opinion scarcely high enough to justify such a serious operation, except in unusual cases with continued relapses, which make the life of the patient such a burden and misery to himself that he will take any risk to get well.

195 Franklin Street.

